# Supplementary material for: Time-series transcriptome analysis identified differentially expressed genes in broiler chicken infected with mixed Eimeria species
Source: Front Genet. 2022 Aug 8;13:886781. doi: 10.3389/fgene.2022.886781 (PMC9393255; doi:10.3389/fgene.2022.886781)
Supplement: Supplementary file 2 [file DataSheet1.ZIP › 7dpi_KEGG.Gsea.1625115961950/KEGG_FOCAL_ADHESION.html]

Details for gene set KEGG\_FOCAL\_ADHESION[GSEA]

|  || Dataset | TMM\_7dpi\_gct\_format\_7dpi\_gct\_format.Class\_4dpi.cls #PC\_versus\_NC.Class\_4dpi.cls #PC\_versus\_NC\_repos |
| Phenotype | Class\_4dpi.cls#PC\_versus\_NC\_repos |
| Upregulated in class | 0 |
| GeneSet | KEGG\_FOCAL\_ADHESION |
| Enrichment Score (ES) | -0.410749 |
| Normalized Enrichment Score (NES) | -1.5347561 |
| Nominal p-value | 8.1599347E-4 |
| FDR q-value | 0.11917492 |
| FWER p-Value | 0.9058 |
Table: GSEA Results Summary

  

Fig 1: Enrichment plot: KEGG\_FOCAL\_ADHESION      
 Profile of the Running ES Score & Positions of GeneSet Members on the Rank Ordered List

  

| SYMBOL | TITLE | RANK IN GENE LIST | RANK METRIC SCORE | RUNNING ES | CORE ENRICHMENT || 1 | TNN | na | 431 | 0.799 | -0.0257 | No |
| 2 | BCAR1 | na | 789 | 0.594 | -0.0478 | No |
| 3 | MYL12B | na | 868 | 0.564 | -0.0468 | No |
| 4 | PAK4 | na | 918 | 0.550 | -0.0436 | No |
| 5 | ITGB5 | na | 1033 | 0.510 | -0.0464 | No |
| 6 | PGF | na | 1105 | 0.483 | -0.0459 | No |
| 7 | HRAS | na | 1342 | 0.420 | -0.0602 | No |
| 8 | VAV3 | na | 1390 | 0.408 | -0.0587 | No |
| 9 | CCND2 | na | 1706 | 0.330 | -0.0808 | No |
| 10 | LAMB4 | na | 1746 | 0.321 | -0.0798 | No |
| 11 | MYL12A | na | 1934 | 0.282 | -0.0918 | No |
| 12 | ITGA3 | na | 2012 | 0.270 | -0.0947 | No |
| 13 | BCL2 | na | 2059 | 0.261 | -0.0951 | No |
| 14 | CAV2 | na | 2253 | 0.229 | -0.1083 | No |
| 15 | PDGFC | na | 2266 | 0.227 | -0.1063 | No |
| 16 | SPP1 | na | 2272 | 0.226 | -0.1037 | No |
| 17 | PPP1CB | na | 2274 | 0.226 | -0.1007 | No |
| 18 | CAV1 | na | 2702 | 0.163 | -0.1346 | No |
| 19 | SHC4 | na | 2866 | 0.143 | -0.1464 | No |
| 20 | ITGA2B | na | 2940 | 0.134 | -0.1508 | No |
| 21 | RAC1 | na | 2998 | 0.126 | -0.1539 | No |
| 22 | PAK5 | na | 2999 | 0.126 | -0.1522 | No |
| 23 | MET | na | 3037 | 0.122 | -0.1537 | No |
| 24 | PPP1CA | na | 3042 | 0.121 | -0.1524 | No |
| 25 | CAPN2 | na | 3045 | 0.121 | -0.1510 | No |
| 26 | PIP5K1C | na | 3125 | 0.111 | -0.1561 | No |
| 27 | FLNA | na | 3217 | 0.099 | -0.1625 | No |
| 28 | LAMB2 | na | 3242 | 0.094 | -0.1632 | No |
| 29 | PDGFD | na | 3313 | 0.085 | -0.1680 | No |
| 30 | ACTN1 | na | 3332 | 0.083 | -0.1684 | No |
| 31 | ITGA11 | na | 3337 | 0.083 | -0.1676 | No |
| 32 | RHOA | na | 3449 | 0.068 | -0.1761 | No |
| 33 | ERBB2 | na | 3936 | 0.011 | -0.2169 | No |
| 34 | BIRC3 | na | 4236 | -0.023 | -0.2419 | No |
| 35 | PDGFB | na | 4266 | -0.026 | -0.2440 | No |
| 36 | PDGFA | na | 4308 | -0.031 | -0.2470 | No |
| 37 | ACTB | na | 4316 | -0.032 | -0.2472 | No |
| 38 | MYL9 | na | 4385 | -0.039 | -0.2524 | No |
| 39 | JUN | na | 4404 | -0.040 | -0.2534 | No |
| 40 | PPP1CC | na | 4419 | -0.041 | -0.2540 | No |
| 41 | LAMA4 | na | 4435 | -0.043 | -0.2547 | No |
| 42 | ACTN4 | na | 4464 | -0.046 | -0.2564 | No |
| 43 | RAP1B | na | 4850 | -0.086 | -0.2877 | No |
| 44 | VWF | na | 4974 | -0.101 | -0.2968 | No |
| 45 | VEGFC | na | 5000 | -0.103 | -0.2975 | No |
| 46 | RAF1 | na | 5142 | -0.116 | -0.3078 | No |
| 47 | COL2A1 | na | 5170 | -0.118 | -0.3085 | No |
| 48 | MAP2K1 | na | 5188 | -0.120 | -0.3084 | No |
| 49 | LAMB1 | na | 5338 | -0.134 | -0.3191 | No |
| 50 | ILK | na | 5351 | -0.135 | -0.3183 | No |
| 51 | CHAD | na | 5390 | -0.138 | -0.3197 | No |
| 52 | ITGA7 | na | 5453 | -0.144 | -0.3230 | No |
| 53 | SHC2 | na | 5501 | -0.149 | -0.3250 | No |
| 54 | MYLK | na | 5505 | -0.149 | -0.3232 | No |
| 55 | ITGA4 | na | 5591 | -0.158 | -0.3283 | No |
| 56 | PPP1R12A | na | 5629 | -0.163 | -0.3292 | No |
| 57 | ITGB3 | na | 5666 | -0.166 | -0.3300 | No |
| 58 | SHC1 | na | 5796 | -0.179 | -0.3385 | No |
| 59 | CDC42 | na | 5837 | -0.184 | -0.3394 | No |
| 60 | ITGA8 | na | 5916 | -0.192 | -0.3434 | No |
| 61 | COMP | na | 5918 | -0.192 | -0.3409 | No |
| 62 | COL1A1 | na | 5931 | -0.194 | -0.3393 | No |
| 63 | ZYX | na | 5978 | -0.199 | -0.3405 | No |
| 64 | THBS4 | na | 6061 | -0.209 | -0.3447 | No |
| 65 | RELN | na | 6066 | -0.209 | -0.3422 | No |
| 66 | RAC3 | na | 6076 | -0.210 | -0.3401 | No |
| 67 | ROCK1 | na | 6268 | -0.231 | -0.3532 | No |
| 68 | SRC | na | 6405 | -0.244 | -0.3614 | No |
| 69 | MAPK8 | na | 6464 | -0.250 | -0.3629 | No |
| 70 | GRB2 | na | 6686 | -0.274 | -0.3779 | No |
| 71 | COL5A1 | na | 6688 | -0.274 | -0.3743 | No |
| 72 | FLT4 | na | 6800 | -0.286 | -0.3798 | No |
| 73 | CRK | na | 6900 | -0.297 | -0.3842 | No |
| 74 | PDGFRA | na | 6935 | -0.300 | -0.3830 | No |
| 75 | AKT3 | na | 7033 | -0.312 | -0.3870 | No |
| 76 | COL3A1 | na | 7109 | -0.320 | -0.3891 | No |
| 77 | CRKL | na | 7144 | -0.324 | -0.3876 | No |
| 78 | PAK2 | na | 7158 | -0.325 | -0.3843 | No |
| 79 | RAPGEF1 | na | 7164 | -0.325 | -0.3804 | No |
| 80 | CCND3 | na | 7194 | -0.329 | -0.3784 | No |
| 81 | ITGB1 | na | 7209 | -0.331 | -0.3752 | No |
| 82 | COL1A2 | na | 7376 | -0.349 | -0.3845 | No |
| 83 | HGF | na | 7414 | -0.353 | -0.3829 | No |
| 84 | PARVB | na | 7477 | -0.359 | -0.3833 | No |
| 85 | MAPK9 | na | 7648 | -0.377 | -0.3926 | No |
| 86 | RAP1A | na | 7657 | -0.378 | -0.3882 | No |
| 87 | COL5A2 | na | 7798 | -0.395 | -0.3948 | No |
| 88 | TLN1 | na | 7840 | -0.400 | -0.3929 | No |
| 89 | ARHGAP35 | na | 7860 | -0.402 | -0.3891 | No |
| 90 | RAC2 | na | 7904 | -0.409 | -0.3872 | No |
| 91 | PDPK1 | na | 8107 | -0.435 | -0.3984 | No |
| 92 | ITGB4 | na | 8156 | -0.440 | -0.3966 | No |
| 93 | DOCK1 | na | 8305 | -0.459 | -0.4029 | No |
| 94 | AKT1 | na | 8399 | -0.472 | -0.4044 | Yes |
| 95 | PARVG | na | 8448 | -0.477 | -0.4021 | Yes |
| 96 | PIK3CG | na | 8478 | -0.481 | -0.3981 | Yes |
| 97 | PTEN | na | 8620 | -0.503 | -0.4032 | Yes |
| 98 | PRKCA | na | 8704 | -0.513 | -0.4034 | Yes |
| 99 | PDGFRB | na | 8726 | -0.517 | -0.3982 | Yes |
| 100 | VEGFA | na | 8816 | -0.526 | -0.3987 | Yes |
| 101 | FN1 | na | 8826 | -0.526 | -0.3924 | Yes |
| 102 | LAMC1 | na | 8856 | -0.531 | -0.3877 | Yes |
| 103 | SOS1 | na | 8869 | -0.533 | -0.3816 | Yes |
| 104 | PAK1 | na | 8943 | -0.541 | -0.3805 | Yes |
| 105 | ITGA9 | na | 9061 | -0.557 | -0.3829 | Yes |
| 106 | PTK2 | na | 9133 | -0.570 | -0.3812 | Yes |
| 107 | ARHGAP5 | na | 9153 | -0.574 | -0.3752 | Yes |
| 108 | THBS1 | na | 9192 | -0.581 | -0.3706 | Yes |
| 109 | CTNNB1 | na | 9201 | -0.583 | -0.3634 | Yes |
| 110 | COL6A3 | na | 9206 | -0.583 | -0.3560 | Yes |
| 111 | KDR | na | 9221 | -0.586 | -0.3493 | Yes |
| 112 | THBS2 | na | 9240 | -0.589 | -0.3429 | Yes |
| 113 | TLN2 | na | 9291 | -0.598 | -0.3391 | Yes |
| 114 | TNR | na | 9381 | -0.611 | -0.3384 | Yes |
| 115 | VEGFD | na | 9472 | -0.627 | -0.3376 | Yes |
| 116 | ITGB6 | na | 9497 | -0.631 | -0.3312 | Yes |
| 117 | COL6A1 | na | 9526 | -0.635 | -0.3250 | Yes |
| 118 | ITGAV | na | 9564 | -0.640 | -0.3196 | Yes |
| 119 | ITGB8 | na | 9583 | -0.642 | -0.3125 | Yes |
| 120 | ROCK2 | na | 9596 | -0.645 | -0.3049 | Yes |
| 121 | PIK3R2 | na | 9597 | -0.645 | -0.2962 | Yes |
| 122 | COL4A1 | na | 9679 | -0.657 | -0.2943 | Yes |
| 123 | COL4A2 | na | 9798 | -0.674 | -0.2952 | Yes |
| 124 | ITGA1 | na | 9831 | -0.679 | -0.2888 | Yes |
| 125 | IGF1R | na | 9854 | -0.683 | -0.2815 | Yes |
| 126 | PRKCB | na | 9867 | -0.685 | -0.2733 | Yes |
| 127 | COL6A2 | na | 9917 | -0.696 | -0.2681 | Yes |
| 128 | RASGRF1 | na | 10171 | -0.746 | -0.2795 | Yes |
| 129 | MYLK3 | na | 10172 | -0.746 | -0.2695 | Yes |
| 130 | MAPK3 | na | 10320 | -0.774 | -0.2715 | Yes |
| 131 | PIK3CB | na | 10335 | -0.777 | -0.2623 | Yes |
| 132 | VCL | na | 10395 | -0.790 | -0.2567 | Yes |
| 133 | COL4A4 | na | 10397 | -0.790 | -0.2462 | Yes |
| 134 | CCND1 | na | 10432 | -0.796 | -0.2384 | Yes |
| 135 | FLNB | na | 10527 | -0.815 | -0.2354 | Yes |
| 136 | ITGA6 | na | 10612 | -0.836 | -0.2313 | Yes |
| 137 | PIK3CD | na | 10707 | -0.855 | -0.2277 | Yes |
| 138 | PIK3CA | na | 10920 | -0.912 | -0.2334 | Yes |
| 139 | PIK3R5 | na | 10941 | -0.918 | -0.2228 | Yes |
| 140 | PIK3R3 | na | 11045 | -0.949 | -0.2188 | Yes |
| 141 | LAMA3 | na | 11104 | -0.965 | -0.2107 | Yes |
| 142 | FLT1 | na | 11183 | -0.995 | -0.2040 | Yes |
| 143 | LAMA1 | na | 11251 | -1.018 | -0.1960 | Yes |
| 144 | LAMA5 | na | 11261 | -1.022 | -0.1830 | Yes |
| 145 | PIK3R1 | na | 11265 | -1.023 | -0.1696 | Yes |
| 146 | PARVA | na | 11300 | -1.037 | -0.1586 | Yes |
| 147 | ITGA2 | na | 11325 | -1.048 | -0.1465 | Yes |
| 148 | LAMB3 | na | 11340 | -1.052 | -0.1336 | Yes |
| 149 | SOS2 | na | 11362 | -1.063 | -0.1211 | Yes |
| 150 | FYN | na | 11389 | -1.076 | -0.1089 | Yes |
| 151 | EGFR | na | 11415 | -1.085 | -0.0965 | Yes |
| 152 | VTN | na | 11634 | -1.193 | -0.0989 | Yes |
| 153 | TNC | na | 11662 | -1.210 | -0.0850 | Yes |
| 154 | PAK3 | na | 11679 | -1.225 | -0.0699 | Yes |
| 155 | BRAF | na | 11724 | -1.264 | -0.0567 | Yes |
| 156 | IGF1 | na | 11859 | -1.426 | -0.0489 | Yes |
| 157 | VAV2 | na | 11979 | -1.860 | -0.0340 | Yes |
| 158 | MAPK10 | na | 12008 | -2.724 | 0.0002 | Yes |
Table: GSEA details [plain text format]

  

Fig 2: KEGG\_FOCAL\_ADHESION      
 Blue-Pink O' Gram in the Space of the Analyzed GeneSet

  

Fig 3: KEGG\_FOCAL\_ADHESION: Random ES distribution      
 Gene set null distribution of ES for **KEGG\_FOCAL\_ADHESION**
